# Does lower extremity alignment during normal stance predict lower limb kinematics and kinetics during drop landings?

**DOI:** 10.1186/s13102-023-00781-6

**Published:** 2023-12-07

**Authors:** Mark G.L. Sayers, Robert L. Buhmann, Tyler J. Collings, Daniel B. Mellifont, Max C. Stuelcken

**Affiliations:** 1 School of Health, University of the Sunshine Coast, Queensland, Australia; 2 School of Health Sciences and Social Work, Griffith University, Queensland, Australia

**Keywords:** Static lower limb alignment, ACL screening

## Abstract

**Background:**

Static lower extremity alignment (LEA) during normal stance has been used clinically as a tool to determine the presence of known anterior cruciate ligament (ACL) risk factors during dynamic tasks. Previous work investigating the relationship between static LEA during normal stance and risk factors for ACL injury is limited by the use of imprecise methods or because it focuses on knee valgus only and no other potentially important variables. The aim of this investigation was to determine the relationships between static LEA and the corresponding LEA during drop landings.

**Methods:**

Forty-one female athletes were recruited for the study (age: 19.8 ± 2.5 years, height: 1.73 ± 0.06 m, mass: 64.03 ± 6.66 kg). Lower limb kinematic data were collected using a 10 camera infrared motion capture system (500 Hz) with retro-reflective markers placed over key anatomical landmarks. This system was linked to two force platforms (1000 Hz) with subsequent three-dimensional kinematic and kinetic data developed using standard software (Visual3D). Following an appropriate warm-up, data collection involved participants standing with their arms partially abducted to record static LEA. This was following by a series of drop landings from a 0.4 m box onto the force platforms. Maximum LEA data during drop landings were then compared with static LEA.

**Results:**

Analyses showed that in comparison to static stance, during landings the anterior tilt of the pelvis decreased while hip abduction and knee internal rotation increased. At best, static LEA variables were moderately correlated (r = -0.51 to 0.58) with peak values measured during drop landings. Additionally, regression analysis did not yield any significant predictors of any key peak hip or knee variables measured during drop landings (p = 0.15 to 0.89).

**Conclusion:**

When combined, the poor relationships observed between kinematics during static LEA and LEA during drop landings calls into question the practice of using static measures to predict LEA during even simple landing tasks. These findings suggest static assessments of LEA may have minimal value as an ACL injury screening tool.

**Supplementary Information:**

The online version contains supplementary material available at 10.1186/s13102-023-00781-6.

## Introduction

Rupture of the anterior cruciate ligament (ACL) is a debilitating injury requiring extensive and expensive rehabilitation with substantial time lost from sport participation [1]. More than 70% of ACL injuries are non-contact in nature [2] and occur typically during landing, sudden deceleration, or cutting tasks [1, 3–5]. Research on risk factors for non-contact ACL injury is extensive and acknowledges that the aetiology is multifactorial in nature [3, 6, 7]. The consensus is that certain movement patterns during landing and/or cutting tasks such as reduced knee flexion [3, 8, 9], excessive knee valgus [3, 10] and internal rotation [11, 12] and excessive frontal plane loading on the knee [10, 13, 14] are all linked to an increased likelihood of ACL injury. Similarly, reduced hip flexion [3, 8, 9], and excessive hip adduction [15] and internal rotation [15–17] during jump landing tasks are all suggested to increase the ACL injury risk. Key consensus statements in this domain highlight that the identification of these modifiable risk factors is an important precursor for the development of effective injury prevention programs [7, 18].

Not surprisingly, a considerable body of literature focuses on the development of efficacious ACL injury screening tools [7, 10, 18–20]. A common approach supports the use of static postural assessments as part of the athlete injury screening processes [21]. Researchers in this domain focus on the links between static lower extremity alignment (LEA) during normal stance [22–28] and the biomechanics associated with ACL injury risk during dynamic tasks. Typically, these studies use inclinometers, goniometry or 2-dimensional images to quantify variables such as anterior pelvic tilt, frontal plane alignment of both the femur (relative to the pelvis) and tibia (relative to the femur), sagittal plane alignment of the tibia (relative to the femur) and bilateral tibia and femoral lengths [23–26]. However, care should be taken when interpreting these data as the inter-tester reliability for some of these measures is often poor (e.g. pelvic tilt [23]) and questions have been raised about the absolute precision of these techniques [23, 27].

To address issues with two dimensional and/or manual data collection techniques, Nilstad, Krosshaug, Mok, Bahr and Andersen [22] used a different approach to assess static LEA, collecting data from static calibration trials performed prior to standard infra-red motion capture. These data were compared subsequently with assessments of 3-dimensional lower limb kinematics during drop landing tasks. Notably, the focus of the research by Nilstad, Krosshaug, Mok, Bahr and Andersen [22] was on the prediction of peak landing knee valgus angles only, concluding that just 11% of the variance in this variable is explained by the combination of increased standing height and static knee valgus. The focus on a single variable by these researchers is limiting, with other researchers noting that static LEA variables are part of the lower limb kinetic chain and do not function independently [25, 29]. In an attempt to address the latter Nguyen, Shultz and Schmitz [26] clustered participants from manual static LEA assessments into those with knee valgus and either internal (C1) or external hip rotation (C3) and those with neutral hip and knee alignment (C2) prior to assessing lower limb kinematics and kinetics during drop landings. They reported participants with static knee valgus alignments (C1 and C3) also demonstrated biomechanics associated with greater risk of ACL injury during drop landings, highlighting the value of using gross LEA measures. In a later study using similar protocols to these researchers Uota, Nguyen, Aminaka and Shimokochi [30] reported significant relationships between measures of static hip internal rotation and knee valgus during drop landings. Importantly these researchers indicated stronger correlations between hip and knee angular motion during the drop landings than between any of these data and static LEA measures. The data collection methods in these studies (i.e. manual assessment of static LEA and electro-magnetic motion capture) rely on different processes to quantify limb position and so some care should be taken when interpreting these data as they may not be comparable. To address the latter, it would appear advantageous to use infra-red motion capture to determine both static LEA and dynamic lower extremity kinematics as systems such as these provide good to excellent within and between test reliability [31].

Accordingly, the purpose of this investigation was to examine the relationships between static LEA and lower extremity kinematics and kinetics during a series of drop landings. To address issues relating to consistency in static LEA assessments, these will be quantified using the static captures that form part of standard motion capture protocols. Analyses will focus on quantifying the relationships between static LEA variables and the key kinematic and kinetic variables during drop landings that are known risk factors for ACL injury [3, 8, 10, 13–17]. The potential findings of this work will add to the literature by assessing relationships between static LEA and lower limb kinematics and kinetics during this fundamental injury movement screening task.

## Methods

Forty-one female athletes provided their written informed consent prior to participating in this investigation (age: 19.8 ± 2.5 years, height: 1.73 ± 0.06 m, mass: 64.03 ± 6.66 kg). Female athletes were chosen as this group is 3–5 times more likely to sustain an ACL injury than males competing in similar sports [32]. All participants were actively involved in sports requiring lower limb power and change of direction ability and competed regularly in state-wide competitions. Inclusion criteria required participants to be both injury free at the time of data collection and to also not have sustained an injury in the preceding 2 months that prevented them from participating in sport for more than 2 weeks. Participants also had to report no joint instability or pain, no history of lower limb or back surgery and were also excluded if they were pregnant. This study was approved by the institutional ethics committee (A/16/878), with all data collection and analysis conducted in accordance with national guidelines and regulations.

Testing occurred during a single session. At the completion of a 10 min self-structured warm-up, which included standard lower body locomotor activities and several practice drop landings, participants were then asked to stand in their “normal stance” for a 3 s data capture with each foot on one of two force platforms (Bertec, Columbus, OH, USA). Data from this static capture were used to define the participants’ static LEA and segmental lengths during the modelling procedures. Following the collection of static data, participants performed a series of six drop landings. The drop landing was chosen as it is one of the most common tasks used to investigate landing biomechanics as a screening tool for ACL injury risk [33]. Standard drop landing test protocols were followed requiring participants to step off a 0.40 m high wooden box, minimising lowering or raising the centre of mass (COM) prior to take-off, before dropping and landing on both feet [33]. There was a rest period of at least 30 s between each trial to minimise the effect of fatigue.

Prior to testing 30 retroreflective spherical markers (10 mm) were placed adjacent to standard anatomical landmarks on the torso, pelvis, thighs, shanks and shoes, with rigid clusters attached bilaterally to the lateral mid shank and thigh [34]. Marker trajectories were recorded at 500 Hz using a 10-camera system (Qualisys Motion Capture System, Gothenburg, Sweden), while ground reaction force (GRF) data were recorded at 1000 Hz. Subsequent 3D motion capture and GRF data were then imported into Visual 3D (C-Motion, Inc., Rockville, MD, USA) where a standard seven segment model of the pelvis and lower limbs was constructed. Marker trajectories were filtered using a low pass, fourth order (zero lag), 12 Hz Butterworth filter, while GRF data were filtered at 38 Hz with cut-off frequencies determined using residual analyses. A global reference system was defined relative to the static capture positions so that the positive Y-axis was directed anteriorly, the X-axis laterally (positive direction to the right) and the positive Z-axis pointing vertically upwards. Joint angles were calculated between adjacent segments with flexion, adduction (and inversion) and internal rotation defined as positive rotations about each segment’s respective X, Y and Z-axes. [34]. External joint moments were calculated with the proximal segment as the resolution coordinate system using standard inverse dynamic procedures and were normalised to body weight (BW) multiplied by height (m). The GRF data were normalised to BW.

Peak values for biomechanical variables were extracted during the landing phase of each trial, with the latter defined as the period from initial ground contact (indicated by a vertical GRF greater than 10 N) to the instance of maximum knee flexion. Variables of particular interest during the landing phase were those linked to risk factors for ACL injury such as large knee valgus angles and moments [10], high peak GRF_vert_ [4], and low knee and hip flexion angles [4]. External joint moments were calculated with the proximal segment as the resolution coordinate system using standard inverse dynamic procedures and were normalised to body weight (BW) multiplied by height (m). The vertical GRF data (GRF_vert_) were normalised to body weight (BW) [9]. All data used in this investigation are available in the associated data set that has been provided with this manuscript.

Shapiro-Wilks tests for normality were conducted for static LEA variables and the equivalent peak values from the landing phase (SPSS Inc., Chicago, IL). Potential differences between static LEA and landing phase data were tested using paired *t-*tests or equivalent non-parametric procedures. This was particularly relevant for the frontal and transverse plane LEA values, as there are obvious increases in hip and knee flexion during landing to facilitate shock attenuation. Effect sizes (Cohen’s *d*) were calculated with the relative magnitude of these effects interpreted as either *trivial* (< 0.20), *small* (0.20 to 0.60), *moderate* (0.60 to 1.20), *large* (1.20 to 2.00), or *very large* (> 2.00) [35]. A priori power analysis (G*Power version 3.1.9.6) indicated that the minimum sample size required to achieve 80% power to detect a *medium* change (*P* = 0.05) in the frontal and transverse plane data was *n* = 34. In an endeavour to compare our data to that developed by Nguyen, Shultz and Schmitz [26] we assessed for potential clustering of static LEA groupings using the same Ward hierarchical clustering techniques adopted by these researchers. However, no clear clusters centring on static knee valgus orientation were apparent in our data and so this procedure was not continued. Pearson Product Moment correlation tests were conducted to determine the relative relationships between these static LEA and drop landing data. The descriptors *small* (0.1), *moderate* (0.3), *large* (0.5), *very large* (0.7) and extremely large (0.9) were used to describe the relative strength of the correlation coefficients [35]. Multiple linear regression analyses were used to determine whether any combination of static LEA variables significantly determined peak values in lower limb alignment during landing. In order to avoid any assumptions of symmetry, all analyses were conducted with respect to side (i.e. Left or Right). Significance for all statistical tests was set at *P* < 0.05 and all data are presented as means ± 1 standard deviation (SD) unless stated otherwise.

## Results

Mean static LEA values indicate that participants’ typical standing posture was characterised by slight anterior pelvic tilt, hip adduction and external rotation and neutral frontal and transverse plane knee alignment (Table 1). The sample population presented with a heterogeneous static LEA, with SD values for hip and knee being 0.7 to 5.3 times the magnitude of the respective means. In comparison to static stance, during the landing phase of the drop landing the pelvis became less anteriorly tilted, the hips became more abducted, and the knees became more internally rotated and moved into valgus. The transverse plane alignment of the hips were the only data to present with non-significant trivial to small changes between the static LEA and landing phase. Participants flexed their hips and knees during landing, with approximately 17° less flexion at their hips than at their knees. Peak GRF_vert_ together with the hip and knee valgus and internal rotation moments during the landing phase are presented in Table 2.

**Table 1:** Differences between the key mean (± 1SD) static LEA values together with similar mean (± 1SD) peak values during the drop landing phase

Segmental alignment ^†^	Side	Static alignment	Peak during landing	*P*	*d*
Pelvic Tilt (°)		-8 (4)	-3 (4)	< 0.001	1.02
Hip – Sagittal plane (°)	Left	-5 (3)	55 (18)	< 0.001	1.84
	Right	-3 (4)	55 (17)	< 0.001	1.82
– Frontal plane (°)	Left	6.9 (4.8)	-3.4 (4.1)	< 0.001	1.51
	Right	7.1 (5.2)	-3.9 (3.3)	< 0.001	1.56
– Transverse plane (°)	Left	-7.0 (5.7)	-8.2 (6.5)	0.379	0.20
	Right	-6.5 (5.6)	-7.6 (5.7)	0.380	0.19
Knee – Sagittal plane (°)	Left	-1 (3)	73 (15)	< 0.001	1.91
	Right	-1 (3)	72 (14)	< 0.001	1.92
– Frontal plane (°)	Left	0.8 (4.0)	-14.3 (5.3)	< 0.001	1.69
	Right	1.0 (4.0)	-13.5 (6.0)	< 0.001	1.64
– Transverse plane (°)	Left	2.6 (6.9)	16.6 (5.6)	< 0.001	1.49
	Right	1.4 (5.4)	16.1 (5.6)	< 0.001	1.60

^†^ Note that positive values represent posterior pelvic tilt, flexion, varus and internal rotation alignments

**Table 2:** Mean (± 1SD) peak values during the landing phase of the drop landing for vertical ground reaction force and hip and knee frontal and transverse plane joint kinetics data

Segmental alignment ^†^	Peak during landing	
	Left leg	Right leg
Peak Vertical Ground Reaction Force (BW)	2.31 (0.55)	2.69 (0.67)
Peak Hip Extension Moment (Nm/kg.m)	0.44 (0.25)	0.44 (0.18)
Peak Hip Abduction Moment (Nm/kg.m)	0.10 (0.11)	0.09 (0.10)
Peak Hip External Rotation Moment (Nm/kg.m)	0.05 (0.03)	0.04 (0.04)
Peak Knee Extension Moment (Nm/kg.m)	1.14 (0.23)	1.14 (0.23)
Peak Knee Valgus Moment (Nm/kg.m)	0.36 (0.15)	0.53 (0.20)
Peak Knee External Rotation Moment (Nm/kg.m)	0.06 (0.05)	0.06 (0.06)

^†^ Note that positive values for hip and knee moments represent extension, abduction/valgus and external rotation

None of the static LEA variables achieved more than *moderate* correlations with the peak values for lower limb alignment recorded during the landing phase (Tables 3 and 4). Similarly, none of the static LEA variables used previously [22] combined significantly during the step-wise multiple regression analyses to predict peak values for any of the peak hip or knee variables during the landing task (P = 0.148 to 0.886).

**Table 3:** Correlation matrix for left leg between key static LEA variables together with similar peak values during the drop landing phase

	Static lower extremity alignment (LEA)				
	Pelvic Tilt	Hip Adduction	Hip Internal Rotation	Knee Varus	Knee Internal Rotation
Peak Hip Flexion	-0.199	0.252	0.236	-0.204	0.016
Peak Hip Adduction	-0.081	0.053	-0.126	0.154	0.111
Peak Hip Internal Rotation	-0.170	0.066	-0.033	-0.227	-0.062
Peak Hip Extension Moment (Nm/kg.m)	0.131	-0.248	0.084	-0.349*	-0.068
Peak Hip Adduction Moment (Nm/kg.m)	0.083	-0.187	-0.266	-0.090	0.093
Peak Hip Internal Rotation Moment (Nm/kg.m)	0.111	-0.160	-0.090	-0.062	-0.020
Peak Knee Flexion	-0.057	-0.217	0.039	-0.297	-0.181
Peak Knee Valgus	-0.092	-0.012	0.065	-0.193	-0.025
Peak Knee Internal Rotation	0.364*	-0.071	-0.102	0.116	0.280
Peak Knee Extension Moment (Nm/kg.m)	-0.214	0.099	0.116	0.124	0.033
Peak Knee Valgus Moment (Nm/kg.m)	0.190	0.097	**-0.336** ^*^	0.232	0.157
Peak Knee Internal Rotation Moment (Nm/kg.m)	-0.017	0.086	0.369*	0.076	-0.039
Peak GRFz (BW)	0.151	0.123	-0.146	**0.411** ^*^	-0.170

* *P* < 0.05

**Table 4:** Correlation matrix for right leg between key static LEA variables together with similar peak values during the drop landing phase

	Static lower extremity alignment (LEA)				
	Pelvic Tilt	Hip Adduction	Hip Internal Rotation	Knee Varus	Knee Internal Rotation
Peak Hip Flexion	-0.185	0.263	0.090	-0.029	-0.167
Peak Hip Adduction	-0.038	0.246	-0.017	0.147	0.235
Peak Hip Internal Rotation	-0.016	-0.048	0.038	-0.080	-0.230
Peak Hip Extension Moment (Nm/kg.m)	0.198	-0.151	-0.112	-0.161	-0.149
Peak Hip Adduction Moment (Nm/kg.m)	-0.310*	0.103	-0.056	-0.237	-0.110
Peak Hip Internal Rotation Moment (Nm/kg.m)	-0.142	0.045	-0.146	-0.091	-0.033
Peak Knee Flexion	-0.035	-0.205	-0.089	-0.168	-0.113
Peak Knee Valgus	-0.004	0.077	0.221	0.052	-0.364*
Peak Knee Internal Rotation	0.121	-0.166	-0.084	0.084	0.135
Peak Knee Extension Moment (Nm/kg.m)	-0.030	0.169	-0.026	0.415**	0.150
Peak Knee Valgus Moment (Nm/kg.m)	-0.006	-0.069	-0.313*	0.041	0.322*
Peak Knee Internal Rotation Moment (Nm/kg.m)	0.316*	-0.036	0.066	0.219	0.231
Peak GRFz (BW)	0.216	0.038	0.159	0.155	0.042

* *P* < 0.05 ** *P* < 0.01

## Discussion

This investigation examined the relationships between static LEA and lower extremity kinematics and kinetics during a series of drop landings. Static postural assessments are a common component of the athlete injury screening processes [21]. In our project, static LEA was quantified using the static captures that form part of standard motion capture protocols, with these data compared directly with LEA data during the drop landings. The specific focus of our analysis was to assess the efficacy of using these static LEA data to predict the key kinematic and kinetic variables during drop landings that are known risk factors for ACL injury [3, 8, 10, 13–17].

Our static LEA hip data shows the typical static posture of this sample of athletic females is characterised by a combination of slight bilateral hip adduction and external rotation. These adduction data are similar to those from earlier studies assessing frontal hip LEA using goniometers and two-dimensional images [23–26]. Similarly, our peak hip kinematic and kinetic data during the drop landing task are comparable to published data from studies using similar protocols and data capture techniques [16, 36]. The first key finding from our analyses concerns the poor relationships between static hip LEA and our lower limb kinematic and kinetic data during landing. This highlights the potential risks involved in using static stance measures to estimate lower limb motion and joint moments during drop landing tasks. Clinicians and trainers should be wary of assuming that an individual’s hip orientation during static stance provides a useful indicator of hip movements during landing tasks.

Our static LEA knee data shows this sample of athletic females presented typically with neutral knee alignment. The reported values are analogous to data from earlier research using similar procedures to determine static frontal plane knee alignment [22]. Our varus/valgus data are considerably smaller than the tibiofemoral angle data reported from frontal plane imagery and/or goniometry [23–26]. Differences between these data (~ 8° less in this study) highlights that care should be taken before comparing studies that use different techniques to measure similar variables. Importantly, participants’ static varus/valgus knee alignment achieved only *small* non-significant correlations with the corresponding peak values during the drop landing task. This finding diverges from research indicating that individuals with static valgus knee alignments present with greater knee valgus during drop landing tasks than those with neutral knee alignments [22, 26]. Differences between our data and those of Nguyen, Shultz and Schmitz [26] are likely to be a function of a combination of the lower level athletes (recreationally active participants in their study versus our well trained athletes) and the exaggeration of these alignments caused by the use of goniometry for assessing static knee varus/valgus alignment [26]. Similarly, the research by Nilstad, Krosshaug, Mok, Bahr and Andersen [22] was based on drop vertical jump landings, tasks that results in different muscle activity and greater ground reaction forces than drop landings [37]. Regardless of these differences, the relatively poor relationships between static knee LEA and our lower limb kinematic and kinetic landing data again questions the practice of using static stance measures to estimate lower limb kinematics and kinetics during drop landing tasks.

The findings in this study also need to be considered in relation to the relative simplicity of the drop landing task itself. Although there is some argument regarding the *representativeness* of this movement to sport specific landing tasks (see review by Collings, Gorman, Stuelcken, Mellifont and Sayers [33]), a drop landing test is easy to perform and allows researchers to minimise the exposure of study participants to potential injury risk factors. However, it would appear that even relatively minor manipulations in task constraints during landings can have profound effects on the usefulness of static LEA testing. For example, the methods for assessing static LEA and landing kinematics and kinetics in this study are almost identical to those presented by Nilstad, Krosshaug, Mok, Bahr and Andersen [22], with the fundamental difference between these investigations centring on their use of a drop vertical jump instead of a drop landing. In contrast to these findings Barrios, Heitkamp, Smith, Sturgeon, Suckow and Sutton [28] show that participants with greater static knee valgus alignment (approximately 3°- 4°) present with greater knee valgus during walking, running and in single leg drop landings. However, the differences in these data between their control and experimental groups was typically the same magnitude as the difference that defined each group (i.e. less than 4°), suggesting these alignments are not altered during these dynamic tasks. In research investigating complex movements such as side-stepping (cutting), Mueske, Abousamra, Katzel, Vandenberg, Pace, Feifer and Wren [38] indicate that static assessments of frontal plane knee alignment do not contribute to the prediction of dynamic knee valgus moments. These researchers highlight that dynamic measures of trunk, pelvic and LEA provide more useful predictors of knee valgus moments during these tasks than any static postural variables. It would appear that the usefulness of static LEA testing as a predictive tool for assessing dynamic landing activities decreases markedly with even relatively small changes in task constraints. While some static LEA variables are associated with some knee and hip kinematic and kinetic variables during drop landing tasks, these relationships are typically *small*. When combined with other research in this domain, we question the efficacy of static LEA assessments for the determination of landing techniques during dynamic tasks.

There are obvious limitations associated with this project. The static position was not standardised between participants, with participants instructed to stand in “normal stance” with feet approximately shoulder width apart. The measures of static LEA in our participants were typical of the research in this domain but may not have been extreme enough to result in changes during the drop landing task [38]. Most importantly, our results may be representative of our test population only and may not be generalizable to other sports, age groups, or competition levels. Static LEA assessment may be a useful tool for determining landing performance for athletes with previous injury, as research highlights differences in the landing mechanics adopted by participants following ACL reconstruction [39]. It is therefore possible that static LEA assessment may be a useful predictive tool with these populations. Similarly, our data are limited to well-trained female athletes and so may not be representative of male and/or untrained athletes [9, 16]. We also focussed primarily on the use of static LEA assessments to predict the key kinematic and kinetic variables during drop landings that are known risk factors for ACL injury. It is possible that static LEA can be used to predict other variables associated with drop landing technique.

## Conclusions

This investigation examined the relationships between static LEA and lower extremity kinematics and kinetics during a series of drop landings in well trained female athletes. Results question the use of static LEA as a predictive tool for the determination of the key kinematic and kinetic variables that are known risk factors for ACL injury during these tasks. Clinicians and trainers should be wary of assuming that an individual’s static stance provides a useful indicator of their lower limb kinematics and kinetics during drop landings.

### Electronic supplementary material

Below is the link to the electronic supplementary material.

Supplementary Material 1

## Data Availability

All data generated or analysed during this study are included in this published article [and its supplementary information files].
